# Allergens stimulate store-operated calcium entry and cytokine production in airway epithelial cells

**DOI:** 10.1038/srep32311

**Published:** 2016-09-08

**Authors:** Amit Jairaman, Chelsea H. Maguire, Robert P. Schleimer, Murali Prakriya

**Affiliations:** 1 Department of Pharmacology, Northwestern University Feinberg School of Medicine, IL 60611, Chicago, USA; 2 Division of Allergy-Immunology, Department of Medicine, Northwestern University Feinberg School of Medicine, IL 60611, Chicago, USA

## Abstract

Aberrant immune responses to environmental allergens including insect allergens from house dust mites and cockroaches contribute to allergic inflammatory diseases such as asthma in susceptible individuals. Airway epithelial cells (AECs) play a critical role in this process by sensing the proteolytic activity of allergens via protease-activated receptors (PAR2) to initiate inflammatory and immune responses in the airway. Elevation of cytosolic Ca^2+^ is an important signaling event in this process, yet the fundamental mechanism by which allergens induce Ca^2+^ elevations in AECs remains poorly understood. Here we find that extracts from dust mite and cockroach induce sustained Ca^2+^ elevations in AECs through the activation of Ca^2+^ release-activated Ca^2+^ (CRAC) channels encoded by Orai1 and STIM1. CRAC channel activation occurs, at least in part, through allergen mediated stimulation of PAR2 receptors. The ensuing Ca^2+^ entry then activates NFAT/calcineurin signaling to induce transcriptional production of the proinflammatory cytokines IL-6 and IL-8. These findings highlight a key role for CRAC channels as regulators of allergen induced inflammatory responses in the airway.

Asthma is a heterogeneous disease arising from a complex interplay of genetic, epigenetic and environmental factors and is believed to affect over 300 million people worldwide^1^,^2^. The disease is characterized by varying levels of bronchoconstriction, airway remodeling and infiltration of a variety of immune cells including eosinophils, basophils and CD4+ T cells, leading to chronic airway inflammation^3^,^4^. Atopy, which refers to allergic sensitization to inhaled allergens such as house dust mites, cockroaches, fungi, pollen and animal dander, is widely associated with asthma^1^,^3^. These allergens activate numerous signaling pathways through a variety of cell surface receptors that include the protease activated receptor type 2 (PAR2), promoting sensitization and culminating in exaggerated inflammatory effector responses^1^,^5^. Elucidation of these signaling cascades is incomplete but remains a major goal of current efforts for identifying new targets for blocking the inflammatory response.

Among the numerous immune and structural cells implicated in the allergen response, airway epithelial cells (AECs) are thought to play an early role in the process through direct interactions with allergens. Located at the interface of the host and the environment, AECs act not only as a mechanical barrier and a first line of defense against inhaled allergens, but also play a vital role in their early recognition and response using pattern recognition receptors such as toll-like receptors (TLRs) and PARs^6^,^7^. Following exposure to allergens, AECs produce inflammatory mediators that activate innate lymphocytic cells, prime dendritic cells to induce Th2 responses from T cells, and induce the production of IL-13 from T cells^3^,^7^ thus helping to shape the multistage immune response seen in asthma. For example, house dust mite (HDM) allergens induce the release of cytokines eotaxin, GM-CSF, CCL2, CCL20, IL-6 and IL-8; cockroach allergens have been shown to induce IL-6, IL-8 and GM-CSF and fungal allergens such as *Alternaria* have been implicated in the release of IL-6, GM-CSF and IL-33 from AECs^8^,^9^,^10^,^11^,^12^,^13^. However, the molecular mechanisms by which these factors are generated and released by AECs are not well-understood. In particular, many if not most allergens induce cytosolic Ca^2+^ elevations in AECs, which in principle could drive transcriptional and enzymatic cascades to induce cytokine production^9^,^12^,^13^,^14^. However, it is not clear whether allergen induced cytosolic Ca^2+^ fluxes arise primarily due to Ca^2+^ release from internal stores or if they also involve activation of specific Ca^2+^ channels on the plasma membrane. Given recent evidence implicating Ca^2+^ signaling in multiple effector functions in AECs^15^,^16^, a clear understanding of how AECs generate Ca^2+^ signals in response to allergens is needed to better understand how cytokine signaling pathways are induced in AECs and to translate this knowledge in the quest for identifying novel targets for therapy.

Store-operated Ca^2+^ release-activated Ca^2+^ (CRAC) channels are the primary mechanism for producing cytosolic Ca^2+^ elevations in many non-excitable cells^17^. CRAC channels are encoded by the *Orai* genes (*Orai*1-3) and activated by the ER Ca^2+^ sensors, STIM1-2^17^,^18^,^19^,^20^. Mechanistically, it is now known that STIM1 and STIM2 sense the [Ca^2+^]_ER_, and, in response to ER Ca^2+^ store-depletion, translocate to the junctional ER to physically interact with Orai channels resulting in CRAC channel gating^17^,^21^. The resulting Ca^2+^ influx regulates a variety of functions including gene expression, cell proliferation and differentiation in many types of cells^17^,^21^.

We and others have shown that CRAC channels are a major mechanism for eliciting Ca^2+^ signals in AECs and are activated in response to PAR2 stimulation^15^,^16^. The ensuing Ca^2+^ elevation regulates the production of key inflammatory cytokines including IL-6, IL-8, and TSLP^15^. Given that many inhaled allergens produce Ca^2+^ elevations in AECs and are thought to mediate their inflammatory effects, at least in part, by stimulating PAR2 receptors^9^,^11^,^12^,^13^, we sought to investigate whether CRAC channels contribute to the downstream response to allergens in bronchial BEAS-2B cells^9^,^11^,^12^,^13^. We report here that cockroach and dust mite allergens activate CRAC channels through stimulation of PAR2 receptors. The ensuing Ca^2+^ signal induces the generation of IL-6 and IL-8. These results highlight an important role for CRAC channels as key regulators for early activation of allergic inflammation in AECs.

## Results

### A screen of allergen extracts reveals insect allergens as activators of store-operated Ca^2+^ entry in bronchial epithelial cells

Many studies have shown that allergen extracts from insects (HDM and cockroach) and fungi (*Alternaria, Aspergillus*) induce cytosolic Ca^2+^ signals in AECs^8^,^13^,^22^. A multitude of factors including activation of PAR2^8^ or other proteolytic receptors and ATP induction^23^ have been implicated in the genesis of these Ca^2+^ signals, yet it is not clear whether they primarily arise due to Ca^2+^ release from internal stores or if they additionally involve Ca^2+^ influx across the plasma membrane. We and others have previously shown that store-operated calcium entry (SOCE) is a major mechanism of Ca^2+^ influx in bronchial epithelial cells and is stimulated by activation of PAR2 receptors^15^,^16^. However, whether allergens can activate CRAC channels in AEC is unknown. We therefore tested the ability of various allergen extracts to activate SOCE in bronchial BEAS-2B cells using fura-2 based Ca^2+^ imaging (Table 1). Specific allergens including HDM, cockroach extracts, chitinase from *Streptomyces griseus* and fungal extracts from *Alternaria* and *Aspergillus* were applied in a Ca^2+^-free medium followed by re-addition of extracellular Ca^2+^ to detect SOCE. This screen revealed that only a subset of allergens tested, limited to extracts from HDM and cockroach allergens, and, to a lesser extent, chitinase from *Streptomyces griseus*, activated store-operated Ca^2+^ signals and Ca^2+^ entry across the plasma membrane in the AECs (Table 1).

### Cockroach extracts induce Ca^2+^ signals in AEC by activating CRAC channels

**Figure 1:**
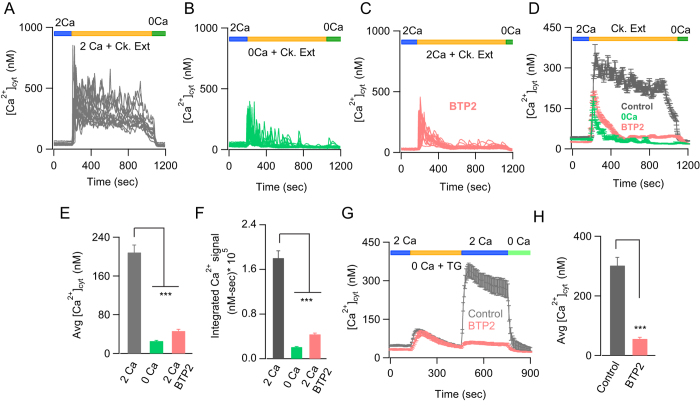
Cockroach allergen extracts activate store-operated CRAC channels in BEAS-2B cells. (**A–C**) [Ca^2+^_i_] imaging showing responses of individual BEAS-2B cells to cockroach allergen extracts (10 μg/mL) administered in (**A**) 2 mM Ca^2+^ ringer’s solution, (**B**) Ca^2+^ free ringer’s solution or (**C**) 2 mM Ca^2+^ ringer’s solution in the presence of the CRAC channel inhibitor BTP2 (500 nM). (**D**) Average [Ca^2+^_i_] response of the individual cells shown in (**A–C**). (**E–F**) Summary of the average rise in [Ca^2+^_i_] 600 seconds after addition of cockroach extract (**E**) and the integral of the [Ca^2+^]_i_ signal during application of the allergen. (**F**). (**G**) Ca^2+^ imaging trace showing SOCE in BAES-2B cells. SOCE was induced by depleting ER Ca^2+^ stores with 1 µM thapsigargin in a Ca^2+^-free Ringer’s solution and readding 2 mM Ca^2+^ following store depletion. Pre-treating cells with BTP2 (500 nM) strongly inhibits SOCE. (**H**) Summary of average rise in cytosolic Ca^2+^ levels 200 seconds after re-addition of 2 mM Ca^2+^ ringer’s following store-depletion. Data are mean ± SEM of 34-47 cells. Representative of 5 independent experiments. **P < 0.01, ***P < 0.001, Ck. Ext, cockroach extract.

There is a strong correlation between sensitization and allergy to inhaled cockroach extracts and the incidence of acute asthmatic attacks^24^,^25^. *In vitro* and *in vivo* studies have shown that extracts from cockroach have proteinase activity and stimulate PAR2 receptors to mediate their inflammatory effects^9^,^10^. Induction of cytosolic Ca^2+^ fluxes in response to cockroach extracts has been shown in alveolar A549 cells^26^, cultured human keratinocytes^27^ and KNRK cells, a rat kidney cell line^10^. However, the pathways mediating these Ca^2+^ fluxes are unknown. We found that administration of cockroach extract to BEAS-2B cells in a 2 mM Ca^2+^ Ringer’s solution produced a biphasic rise in cytoplasmic Ca^2+^: a rapid initial spike followed by sustained Ca^2+^ signals that lasted more than 10 minutes (Fig. 1A). In most cells, the sustained component of the Ca^2+^ response consisted of an elevated baseline with an oscillating component superposed on the baseline. The sustained signals elicited by cockroach extract were almost completely abolished in a Ca^2+^ free Ringer’s solution, suggesting that Ca^2+^ influx across the plasma membrane was needed for this Ca^2+^ signal (Fig. 1B). Moreover, the CRAC channel inhibitor, BTP2, significantly inhibited both the plateau Ca^2+^ signals as well as the oscillating component (Fig. 1C), indicating that the sustained Ca^2+^ signals arise from the opening of CRAC channels. Fig. 1A–C show traces from individual cells, whereas the average [Ca^2+^]_i_ changes and the integrated area under the curve during the time period of allergen treatment are summarized in Fig. 1D–F. To confirm the inhibitory effects of BTP2 on CRAC channel activation in BEAS-2B cells more directly, we activated SOCE using thapsigargin, a SERCA pump inhibitor that irreversibly depletes ER Ca^2+^ stores. SOCE was significantly inhibited by BTP2 at the same concentration that was used to inhibit allergen induced Ca^2+^ signal (Fig. 1G,H). Based on these results, we conclude that cockroach extracts induce long lasting Ca^2+^ signals in bronchial epithelial cells by activating SOCE through CRAC channels.

### STIM1 and Orai1 mediate cockroach extract induced Ca^2+^ entry in bronchial epithelial cells

**Figure 2:**
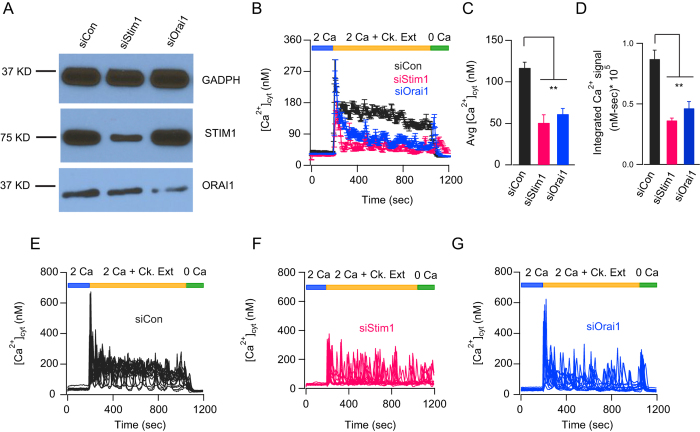
STIM1 and Orai1 mediate Ca^2+^ entry evoked by cockroach allergen extracts. (**A**) Western blot showing STIM1 and Orai1 expression in BEAS-2B cells and the effects of siRNA knockdown of *STIM1* or *Orai1*. (**B**) Ca^2+^ traces showing the effects of siRNA knockdown of STIM1 and Orai1 on cockroach allergen-induced Ca^2+^ signals. A scrambled siRNA sequence was used as control. (**C,D**) Summary of average cytosolic Ca^2+^ levels 798 seconds after addition of cockroach allergen extract (8 μg/mL) (**C**) and the integral [Ca^2+^] signal following addition of cockroach extract (**D**). (**E–G**) Ca^2+^ imaging traces of individual cells treated with either a scrambled control siRNA (**E**) or siRNA against *Stim1* (**F**) or *Orai1* (**G**) showing effects of the knockdown on cockroach allergen-induced Ca^2+^ signals. Data are mean ± SEM of 29–38 cells. Representative of 3 independent experiments. **P < 0.01.

We have previously shown in AECs that SOCE is mediated by the CRAC channel proteins, STIM1 and Orai1^15^. Knockdown of STIM1 and Orai1 using siRNA reduced expression of these proteins (Fig. 2A and Supplementary Fig. S1) and significantly reduced the amplitude of Ca^2+^ elevation induced by cockroach extract (Fig. 2B). Both the average amplitude of Ca^2+^ signal and the integrated Ca^2+^ signal over time was significantly attenuated in si*STIM1* and si*Orai1* treated cells (Fig. 2C,D). Knockdown of STIM1 by si*Stim1* showed good specificity and did not have any effect on STIM2 expression (Supplementary Fig. S1). Interestingly, analysis of single cell Ca^2+^ responses revealed that a proportion of cells in both *siSTIM1* and *siOrai1* treated samples showed Ca^2+^ oscillations (Fig. 2F,G). It is likely that, given the incomplete knockdown of STIM1 and Orai1 by siRNA (Fig. 2A), these oscillatory signals are mediated by the residual CRAC channel machinery. In contrast, cells treated with the siRNA control showed a more heterogeneous response with individual cells showing a sustained increase in Ca^2+^ signal with oscillatory Ca^2+^ signals riding on top of the elevated baseline, which accounted for the higher average Ca^2+^ response (Fig. 2E). We also note that the average Ca^2+^ elevation in response to cockroach extracts seen in *siControl* treated cells was lower than in untransfected control cells (Fig. 1D) likely due to the cell stress induced by cell transfection with lipofectamine. Taken together, these results demonstrate that STIM1 and Orai1 make essential contributions to the Ca^2+^ elevations in AECs following exposure of the cells to cockroach extracts.

### Dust mite extracts induce Ca^2+^ signals in AECs by activating CRAC channels

**Figure 3:**
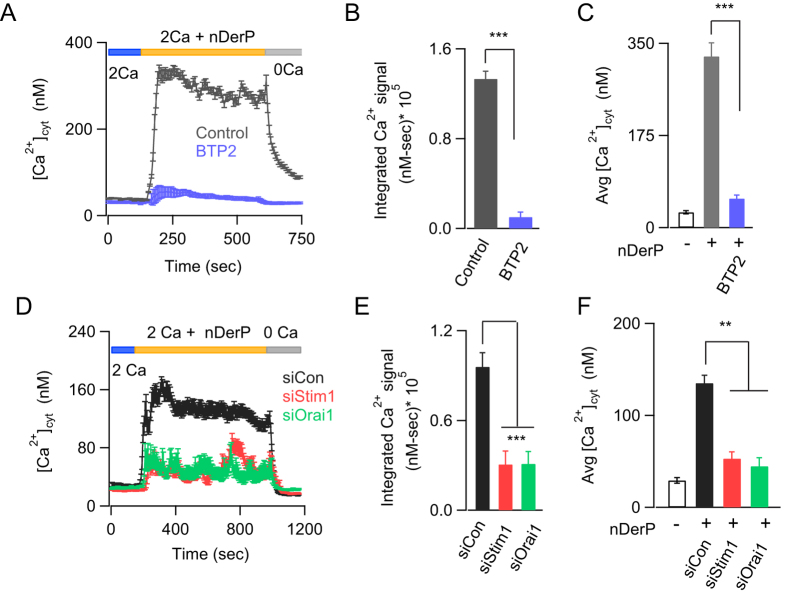
Dust mite allergen extracts activate CRAC channels in BEAS-2B cells. (**A**) HDM extract (nDerP, 18 μg/mL) induces a cytosolic Ca^2+^ signal that is blocked by BTP2. (**B,C**) Summary of the average rise in [Ca^2+^_i_] 200 seconds after addition of HDM extract (**B**) and the integral Ca^2+^ signal (**D–F**) Knockdown of STIM1 or Orai1 inhibits nDerP induced Ca^2+^ signals. (**D**) Average trace showing the effects of siRNA mediated knockdown of *STIM1* and *ORAI1* on HDM allergen-induced Ca^2+^ signals. Summary of (**E**) average cytosolic Ca^2+^ levels 540 seconds after addition of dust mite allergen extract and (**F**) integrated Ca^2+^ signal. Data are mean ± SEM of 32–57 cells. Representative of 3–5 independent experiments. **P < 0.01, ***P < 0.001, nDerP, dust mite extract.

Previous studies have shown that exposure to HDM can trigger allergic inflammation in asthmatic patients^1^,^28^,^29^. Components of HDM, including Der p1, Der p3 and Der p9 exhibit proteolytic activity that leads to activation of PAR2 receptors, which in turn plays a critical role in mediating the inflammatory effects of HDM^8^,^30^. HDM has also been shown to activate Ca^2+^ signals in airway epithelial cells in both primary epithelial cells and cell lines, and this is believed to occur through both PAR2-dependent and -independent mechanisms^8^,^30^,^31^. However, whether HDM can activate CRAC channels has not been studied. When administered in a Ca^2+^-free Ringer’s solution, HDM induced only a transient Ca^2+^ signal indicating that the extract causes Ca^2+^ release from internal stores (Supplementary Fig. S2). In the presence of extracellular Ca^2+^, however, HDM extracts activated a sustained Ca^2+^ signal in BEAS-2B cells that was inhibited by the CRAC channel inhibitor BTP2 (Fig. 3A–C, Supplementary Fig. S2). Further, knockdown of the CRAC channel proteins STIM1 and Orai1 significantly abrogated the average sustained Ca^2+^ signals seen in response to HDM (Fig. 3D–F). These results indicate that HDM allergens mobilize cellular Ca^2+^ elevations in bronchial epithelial cells by depleting ER Ca^2+^ stores and activating CRAC channels encoded by STIM1 and Orai1.

### Insect allergens mobilize Ca^2+^ signals by activating PAR2 receptors

**Figure 4:**
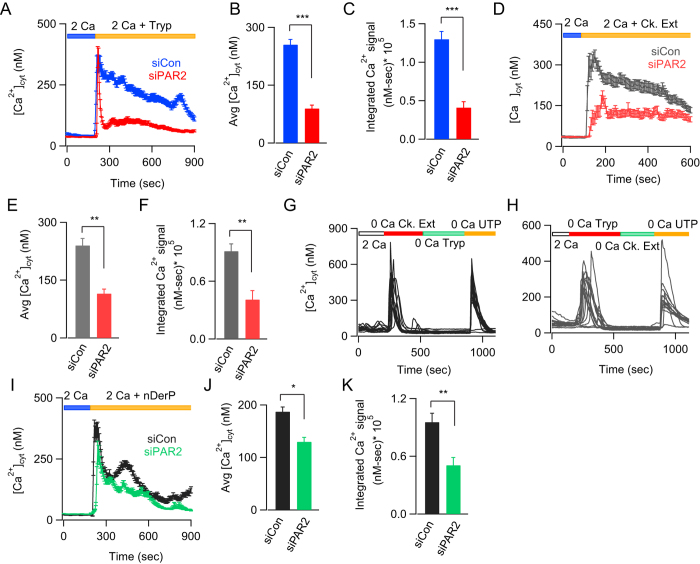
Cockroach and dust mite allergens evoke Ca^2+^ signals through PAR2 activation. (**A–C**) The PAR2 agonist type IX trypsin (50 nM) mobilizes [Ca^2+^]_i_ in BEAS-2B cells. This [Ca^2+^]_i_ elevation is suppressed by siRNA knockdown of PAR2. (**B**). Summary of the average cytosolic [Ca^2+^] rise 150 seconds after addition of trypsin. Summary of the integral Ca^2+^ signal following application of trypsin. (**D–F**) Ca^2+^ responses to cockroach allergen (8 μg/mL) in cells treated with siControl or siPAR2. (**E**) Summary of the average [Ca^2+^] rise 100 seconds after addition of cockroach extract. Summary of the integrated Ca^2+^ signal following application of the extract. (**G,H**) Paired application of the PAR2 agonist trypsin and cockroach allergen shows that prior activation of PAR2 by trypsin diminishes the subsequent allergen response and *vice versa*. Subsequent response to a non-PAR2 agonist, UTP, is unaffected suggesting that the lack of response in the paired application is not due to depletion of ER-stores but rather due to receptor desensitization. (**I–K**) Response to dust mite allergens is partially inhibited by knockdown of PAR2. Mean ± SEM of 21–43 cells, 2 experiments. *P < 0.05, **P < 0.01; Tryp, trypsin.

Both HDM and cockroach allergens have been shown to activate PAR2 receptors on airway epithelial cells^1^,^9^,^30^,^31^. Whether this is the primary mechanism by which cellular Ca^2+^ signals are generated remains a contentious issue, with evidence for both PAR2 dependent and independent mechanisms^8^,^30^. We therefore studied the effect of siRNA mediated PAR2 receptor knockdown on allergen induced Ca^2+^ influx. Cytosolic Ca^2+^ elevations in response to type IX trypsin, a well characterized PAR2 agonist, was strongly inhibited in the siRNA treated cells, confirming knockdown of PAR2 in these cells (Fig. 4A–C). Importantly, Ca^2+^ influx seen in response to cockroach allergens was also significantly inhibited in si*PAR2* treated cells, indicating that cockroach extracts induced Ca^2+^ signals by activating PAR2 receptors (Fig. 4D–F). This conclusion is further supported by the Ca^2+^ responses seen following paired application of the allergen and the PAR2 specific agonist, trypsin. Following application of the cockroach extract, administration of trypsin failed to elicit a Ca^2+^ signal, suggesting that trypsin and cockroach extract activate the same signal transduction pathway, and prior desensitization of the PAR2 receptor or immediate downstream signaling attenuates the response to a second challenge to PAR2 (Fig. 4G). By contrast, Ca^2+^ mobilization in response to P2Y receptor activation by UTP (therefore PAR2-independent) was unaffected following prior treatment with the allergen. Likewise, pre-application of the PAR2 agonist trypsin impaired a subsequent Ca^2+^ response to cockroach allergen but not to UTP. (Fig. 4H). These results are consistent with the interpretation that cockroach allergens activate PAR2 receptors in AECs. However, in contrast to the effects of the cockroach extracts, knockdown of PAR2 elicited only modest inhibition of the Ca^2+^ response to dust mite extract (Fig. 4I–K). This result suggests that the Ca^2+^ response to dust mite extracts is mediated by both PAR2-dependent as well as independent mechanisms.

### Ca^2+^ responses to fungal and bacterial allergens

**Figure 5:**
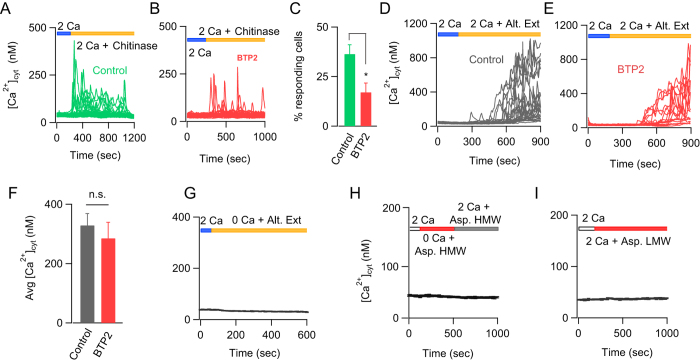
Effect of bacterial and fungal allergens on Ca^2+^ signaling in BEAS-2B cells. (**A–C**) Chitinase from *Streptomyces griseus* (30 μg/mL) induces oscillatory Ca^2+^ signal that is blocked by BTP2. (**D–G**) Effect of *Alternaria alternata* extracts on Ca^2+^ signaling in AECs (**D**) *Alternaria alternata* extracts (30 μg/mL) induce cytosolic Ca^2+^ elevations in AECs that is not blocked by BTP2 (**E**). (**F**) Summary of average Ca^2+^ rise at the 900 second time point. (**G**) *Alternaria alternata* extracts (30 μg/mL) do not cause ER store-release. (**H,I**) Extracts from *Aspergillus fumigatus* high molecular weight fraction (10 μg/mL) (**H**) and low molecular weight fraction (10 μg/mL) (**I**) fail to induce Ca^2+^ signals in BEAS-2B cells. (N = 17–34 cells, Mean ± SEM of 3 experiments). *P < 0.05, **P < 0.01 *P < 0.05, Alt, *Alternaria alternata*; Asp, *Aspergillus fumigata*; HMW, high molecular weight fraction; LMW. Low molecular weight fraction.

The ability of cockroach and dust mite extracts to stimulate SOCE led us to next consider whether this Ca^2+^ influx pathway might be a common feature of other allergenic pathways. *Chitinase* enzyme from fungal and insect sources has been implicated in airway inflammation and elevated expression of a mammalian *chitinase* enzyme has been noted in mouse models of asthma and in allergic asthma in humans^22^. Moreover, Hong *et al.* have noted that *Chitinase* stimulates Ca^2+^ flux in airway epithelial cells through a mechanism likely involving PAR2 receptors^22^. We found that chitinase extracts from *Streptomyces griseus* produced oscillatory Ca^2+^ signals in a significant fraction of BEAS-2B cells (Fig. 5A). These Ca^2+^ signals were inhibited by exposing cells to BTP2, suggesting that, like cockroach and HDM extracts, chitinase activates CRAC channels in AECs (Fig. 5A–C).

Allergens derived from the fungus *Alternaria alternata* have been shown to trigger a Th2 type response through the release of IL-33 from airway epithelial cells in a Ca^2+^ dependent manner^32^,^47^. *Alternaria* also induces the production of IL-6, IL-8 and GM-CSF from AEC^13^. We found that following treatment with *Alternaria* extracts, BEAS-2B cells showed a slow but progressive increase in their [Ca^2+^]_i_ levels at concentrations of 30 μg/mL or above (Fig. 5D). However, this increase was not affected by pre-treatment with BTP2, ruling out involvement of CRAC channels in this process (Fig. 5E,F). Furthermore, *Alternaria* extracts did not evoke release of Ca^2+^ from internal stores (Fig. 5G). We did not observe concomitant reduction of fura 340 and 380 signal following addition of the fungal extracts, ruling out the possibility of proteolytic cell damage. These results indicate that the slow [Ca^2+^]_i_ rises evoked by *Alternaria* extracts do not involve CRAC channels.

Another fungus that is commonly associated with inflammatory lung diseases including asthma, allergic sinusitis, bronchopulmonary aspergillosis, and chronic eosinophilic pneumonitis, is *Aspergillus fumigatus*^33^. Although some studies have implicated cross-talk between TLRs and PAR2 receptors in the response to this fungus, the basic mechanisms by which *Aspergillus fumigatus* triggers airway inflammation remain largely unknown. In our tests, neither low nor high molecular weight fractions of extracts from *Aspergillus fumigatus* induced Ca^2+^ signals in bronchial BEAS-2B cells (Fig. 5H,I). Thus, the ability of *Aspergillus fumigatus* extracts to modulate signaling pathways in AECs including inhibition of Jak-Stat signaling^33^ is likely not mediated by CRAC channels. Overall, these results suggest that the activation of Ca^2+^ influx through CRAC channels is confined to a specific subset of insect allergens that include cockroach, dust mites, and chitinase enzyme.

### Dust mite and cockroach allergens induced generation of IL-6 and IL-8 through activation of CRAC channels

**Figure 6:**
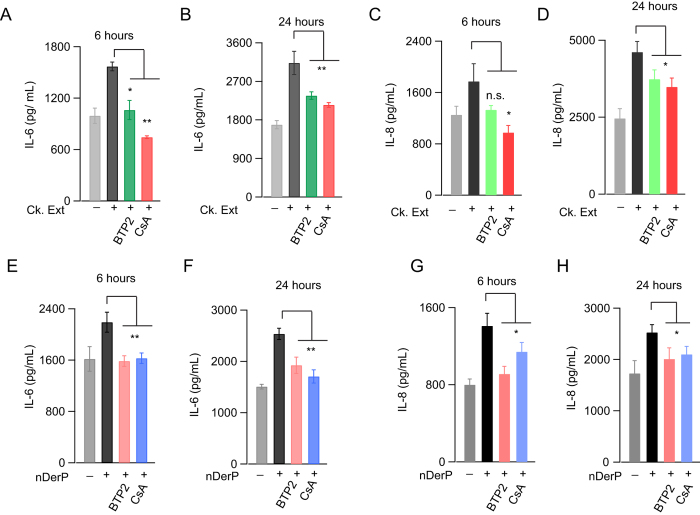
Cockroach and dust mite allergens induce IL-6 and IL-8 in a CRAC channel dependent manner. (**A–H**) BEAS-2B cells were treated with cockroach (10 μg/mL) or and dust mite (18 μg/mL) allergen extracts for 6 or 24 hours. Cells were pre-treated with the CRAC channel inhibitor, BTP2 (500 nM) or the calcineurin inhibitor cyclosporine **A** (CsA, 500 nM) for 45 minutes before allergen treatment. After allergen treatment, cell culture supernatants were collected and examined for IL-6 and IL-8 using ELISA. Mean ± SEM of 2–3 experiments with three replicates for each expeirment. *P < 0.05, **P < 0.01.

An important consequence of allergen sensing by the airways is the induction of pro-inflammatory mediators such as IL-6 and IL-8, which leads to the recruitment of various immune cells to the airway^9^,^10^,^12^. IL-8 plays an important role in the recruitment of neutrophils to the site of airway injury whereas IL-6 is a pleiotropic cytokine that is critical for B cell differentiation as well as T cell activation^34^. We found that exposure of BEAS-2B cells to cockroach allergens or HDM extracts resulted in the induction of IL-6 and IL-8 both at 6 and 24 hour time points (Fig. 6A–H). The induction of these cytokines was abolished by the CRAC channel antagonist, BTP2, indicating that Ca^2+^ entry through CRAC channels is essential for the generation of these cytokines (Fig. 6A–H). This result is consistent with our previous report demonstrating that activation of PAR2 receptors leads to induction of IL-6 and IL-8 in a CRAC channel dependent manner^15^. Moreover, cyclosporine A, a calcineurin inhibitor, impaired the generation of IL-6 and IL-8 following challenge by allergens, indicating that calcineurin/NFAT signaling is critical for the induction of IL-6 and IL-8 by allergens (Fig. 6A–H)^35^. Together, these results indicate that insect allergens stimulate the production of IL-6 and IL-8 via NFAT dependent gene expression that is driven by Ca^2+^ entry through CRAC channels.

## Discussion

Interactions between common allergens found in ambient air such as insect (house dust mites, cockroach) or fungal (*Aspergillus, Alternaria*) allergens and the airway epithelium underlie the development of airway inflammation seen in allergic diseases like asthma^36^,^37^. The biological effect of these allergens is mediated, in part, by proteolytic activity contained within them, which produces epithelial cell damage and activates protease activated receptors to trigger signaling cascades that lead to production of several key inflammatory mediators from AECs^5^. A key signaling event in allergen induced cell signaling is the elevation in cytosolic Ca^2+^, which has been proposed to occur through both PAR2 dependent and independent pathways^10^,^11^,^12^,^30^. However, the specific Ca^2+^ entry pathways that mediate allergen-evoked Ca^2+^ signals have not been determined. In this study, we show that insect allergens derived from cockroach and dust mite extracts activate CRAC channels in bronchial epithelial cells. CRAC channel activation occurs, at least in part, through stimulation of PAR2 receptors and mediates an important role in the induction of the inflammatory modulators IL-6 and IL-8.

Our primary finding is that cockroach and dust mite allergens induce sustained and oscillatory Ca^2+^ signals in bronchial BEAS-2B cells by activating CRAC channels. Both pharmacological inhibition by BTP2 and knockdown of the canonical CRAC channel proteins STIM1 and Orai1 significantly abrogated insect allergen induced Ca^2+^ signals (Figs 2 and 3). For both allergens, while the sustained component of the Ca^2+^ signal was completely inhibited in Ca^2+^ free buffer (Fig. 1B and data not shown), some residual Ca^2+^ influx persisted in cells in which STIM1 and Orai1 were knocked down (Figs 2F and 3D). This is most likely due to the incomplete knockdown of CRAC channel proteins as seen in the Western blot (Fig. 2A). However, we cannot rule out that the possibility that other CRAC channel proteins (STIM2, Orai2, 3) also make some contribution to the allergen-induced Ca^2+^ signals. Interestingly, while the average [Ca^2+^]_i_ rise following stimulation with cockroach extracts was lower in si*STIM1* and si*Orai1* treated cells, a fraction of these cells showed Ca^2+^ oscillations (Fig. 2F,G). The specific nature of Ca^2+^ signals is often determined by complex interactions between the agonist, agonist receptor, IP_3_ receptors and Ca^2+^ channels^38^. It is possible that the reduced Ca^2+^ influx in the si*STIM1* and si*Orai1* treated cells fundamentally affected feedback to IP_3_ receptors and changed the nature of Ca^2+^ signals to the oscillatory type. It would be interesting to test if the sustained Ca^2+^ signals seen in response to cockroach and dust mite allergens become oscillatory in nature when the concentration of the allergens is reduced in the external media, as has been shown for other agonists like ATP^39^. If true, this could have important implications for downstream signaling. For example, depending on the concentration of inhaled allergens in the airway, AEC might produce either oscillatory or sustained Ca^2+^ signals, with each producing a distinct biological response.

Several studies have established a role for PAR2 receptors in the induction of Ca^2+^ signals in response to cockroach and dust mite allergens^9^,^10^,^12^,^40^. While components of dust mite allergens such as Der p3 and Der p5 induce Ca^2+^ signals in kidney and alveolar epithelial cell lines through PAR2 receptors, other components such as Der p1 do not activate Ca^2+^ signals^8^,^30^. We found that knockdown of PAR2 receptors significantly inhibited Ca^2+^ signals in response to both cockroach and dust mites, though the inhibition of Ca^2+^ signal was incomplete, likely due to incomplete knockdown of the PAR2 protein (Fig. 4). However, given that the dust mite extract likely contains a combination of several serine proteases, and possibly many other undefined proteins, it is possible that additional PAR2 independent mechanisms also mediate the observed elevation of cellular Ca^2+^ signals. Future studies that examine the Ca^2+^ responses to specific purified or recombinant dust mite proteases (e.g., Der p1, 3, 5 and 9) will help to discern the contributions of precise components of the dust mite extracts to the observed Ca^2+^ signals.

Interestingly, we failed to detect the involvement of CRAC channels in the Ca^2+^ elevation evoked by extracts of the *Alternaria* fungus. A previous study that used a high concentration of *Alternaria* extracts (200 μg/mL) found that the extracts cause Ca^2+^ elevations acting through the autocrine stimulation of purinergic receptors by ATP^23^. Here, we employed a lower concentration of the extract (30 μg/mL) and found that while the extract induced Ca^2+^ elevations in the presence of extracellular Ca^2+^, no response occurred in Ca^2+^-free Ringer’s buffer, arguing against activation of purinergic receptors, at least in the concentration range we tested. Moreover, the observed Ca^2+^ influx seen in the presence of extracellular Ca^2+^ was not dependent on CRAC channels as the CRAC channel inhibitor BTP2 had no effect. These results indicate that *Alternaria* evokes Ca^2+^ influx through other Ca^2+^ influx pathways whose identity remains to be determined.

In conclusion, we provide evidence showing that bronchial epithelial cells sense cockroach and dust mite allergens through the activation of cell surface PAR2 receptors, which in turn leads to the opening of store-operated CRAC channels. The ensuing Ca^2+^ signal is known to play an important role in the induction of the cytokines IL-6 and IL-8 through an NFAT dependent mechanism. These results demonstrate that CRAC channels may have a central role as effectors of allergen signaling in the airway epithelium.

## Methods

### Cells and media

The bronchial epithelial cell line BEAS-2B, a kind gift from Curtis C. Harris (National Cancer Institute), was cultured in DMEM/F12 medium (CellGro) containing 5%FBS (Hyclone), 50 U/mL penicillin and 50 mg/mL Streptomycin and was maintained at 37 °C and in 5% CO_2._ Cells from passage 44-51 were used for experiments.

### Plasmids and transfections

siRNAs used to down regulate STIM1, STIM2, Orai1 and PAR2 protein expression along with scrambled siRNA negative control were purchased from Ambion, Life Technologies (SilencerSelect predesigned siRNA). siRNA constructs were transfected into BEAS-2B cells using Lipofectamine2000 (Invitrogen) according to manufacturer instructions. Cells were used for experiments 48–72 hours after transfection.

### Reagents and chemicals

The standard extracellular Ringers solution had the following composition (in mM): 150 NaCl, 4.5 KCl, 10 D-glucose, 1 MgCl_2_, 2 CaCl_2_ and 5 Na-HEPES. pH was adjusted to 7.4 using NaOH. For the Ca^2+^ free Ringers solution, CaCl_2_ was excluded from the above composition and MgCl_2_ was increased to 3 mM. Stock solutions of BTP2, thapsigargin (TG) and cyclosporinA (CsA) were made in DMSO. PAR2-agonist type IX trypsin (Sigma) was constituted in water. Dust mite allergen extract (nDer p) was from Indoor Biotechnologies Inc, Charlottesville, VA. Cockroach extract was purchased from HollisterStier Allergy, Spokane, WA. CRAC channel inhibitor BTP2 was from Millipore, Billerica, MA. All other compounds were from Sigma Aldrich, St. Louis, MO.

### Intracellular Ca^2+^ measurements

BEAS-2B cells were grown on poly-L lysine coated glass bottom dishes (MatTek Corp, Ashland, MA). Cells were loaded with 2.5 μM Fura-2 AM (Thermo Scientific Fisher, Waltham, MA) in DMEM/F12 and 5% FBS culture media for 40 minutes at room temperature. Excess fura2 was washed off and cells were incubated in media for an additional 10 minutes before imaging. Single cell [Ca^2+^]_i_ measurements were done according to the protocol described previously^41^. Image acquisition and analysis was performed using IPLab (Scanalytics, Rockville, MD, USA) and Slidebook. For analysis, regions of interest were drawn around single cells and following background subtraction, the *F*_*340*_*/F*_*380*_ intensity ratio was obtained as a function of time. The ratios were converted to [Ca^2+^]_I_ using the formula


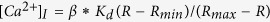


where R is the *F*_*340*_*/F*_*380*_ fluoresce intensity ratio and R_max_ (9.645) and R_min_ (0.268) were determined by *in-vitro* calibration of FURA2^42^. β (20.236) was determined from the *F*_min_/*F*_max_ ratio at 380 nm and *K*_*d*_ is the apparent dissociation constant of fura-2 binding to Ca^2+^ (135 nM).

### Western blots

BEAS-2B cells were cultured in 6-well plates. At 70% confluency, cells were washed with cold PBS and lysed in a solution containing 150 mM NaCl, 50 mM Tris, 1% Triton-X-100, 0.1% SDS and 1x Protease Inhibitor Cocktail (Sigma) for 45 minutes. Cell lysates were obtained using a cell scraper, lysates were spun down at 4 °C for 30 minutes and supernatants were collected and stored at −80 °C. For Western blotting, samples were heated to 99 °C for 5 minutes in Laemmli Sample Buffer (Bio-Rad) containing 0.1% β-mercaptoethanol, run on 10% SDS-PAGE gels, and transferred to nitrocellulose membrane. Orai1, STIM1, and STIM2 proteins were detected using an affinity purified polyclonal antibodies and peroxidase labelled secondary antibodies^43^,^44^.

### Analysis of cytokine secretion

BEAS-2B cells were cultured on 24 well plates in DMEM/F12 media with 5% FBS. 24–48 hours later, cells were pre-treated with CRAC channel inhibitor BTP2 (500 nM) or calcineurin inhibitor Cyclosporin A (500 nM) for 45–60 min before being stimulated with dust mite and cockroach allergens for 6 or 24 hours. Supernatants were collected and stored at −80 deg. C. Levels of inflammatory mediators IL-6 and IL-8 was measured using ELISA kits (RayBiotech for IL-6, and LifeTechnologies for IL-8).

### Data analysis

Average cytosolic Ca^2+^ traces and bar graphs summarizing the data are reported as mean ± SEM. For data sets involving more than two groups, initial statistical analysis was performed using ANOVA with a confidence interval of 5%. This was followed by two-tailed paired student t-test for comparing different treatment conditions within the set.

## Additional Information

**How to cite this article**: Jairaman, A. *et al.* Allergens stimulate store-operated calcium entry and cytokine production in airway epithelial cells. *Sci. Rep.* **6**, 32311; doi: 10.1038/srep32311 (2016).

## Supplementary Material

Supplementary Information

## Figures and Tables

**Table 1:** Effect of insect and fungal allergen extracts on SOCE in BEAS-2B cells.

Allergen	Concentration tested (μg/mL)	Known Receptor	References	SOCE activation?
Cockroach extract	5–10	PAR2	9, 10	++
Dust mite extract	12–18	PAR2, other protease receptors	8, 30, 45	++
Der p1	5–20	PAR2	8	−
*Aspergillus fumigatus*	3.2–10	PAR2	46	−
*Alternaria alternata*	20–30	PAR2, ATP	32, 23, 47	−
Chitinase from *Streptomyces griseus*	20–30	PAR2	22	+

BEAS-2B cells were exposed to the indicated allergen extracts in a Ca^2+^ free Ringer’s buffer followed by readdition of 2 mM Ca^2+^ Ringer’s solution to induce SOCE. The amplitude of cytosolic Ca^2+^ rise, [Ca^2+^]_i_ was examined following addition of 2 mM Ca^2+^ by Ca^2+^ imaging using Fura2 dye. Cells were considered responders if the [Ca^2+^]_i_ elevation was >2 × SEM above the resting [Ca^2+^]_i_. (+, Avg. response <2x above baseline [Ca^2+^]_i_, ++, Avg. response >2x above baseline [Ca^2+^]_i_).
