# Supplementary material for: Allergens stimulate store-operated calcium entry and cytokine production in airway epithelial cells
Source: Sci Rep. 2016 Sep 8;6:32311. doi: 10.1038/srep32311 (PMC5015156; doi:10.1038/srep32311)
Supplement: Supplementary Information [file srep32311-s1.pdf]

Supplementary Information

Allergens stimulate store-operated calcium entry and cytokine production in airway epithelial cells

Amit Jairaman[1], Chelsea H. Maguire[1], Robert P. Schleimer[2] and Murali Prakriya[1,*]

[1]Department of Pharmacology, [2]Division of Allergy-Immunology, Department of Medicine, Northwestern University Feinberg School of Medicine, Chicago, IL 60611

(* address correspondence to m-prakriya@northwestern.edu)

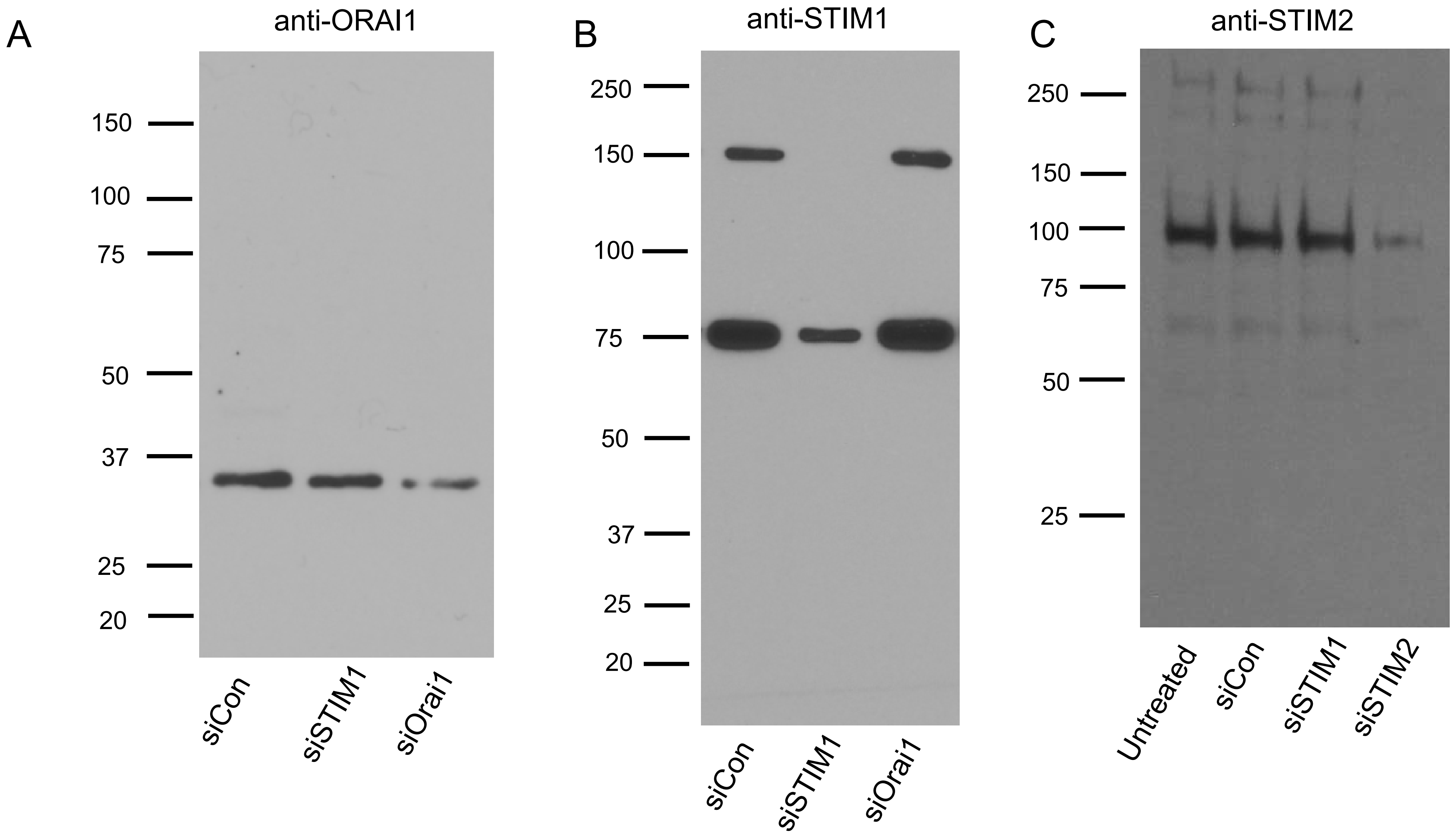

Supplementary Figure S1: **Expression of CRAC channel proteins in BEAS2B cells.** Western blots showing expression of Orai1 (A), STIM1 (B) and STIM2 (C) and knockdown by siRNAs against these proteins or by a scrambled control (siCon) siRNA. See Methods for details.

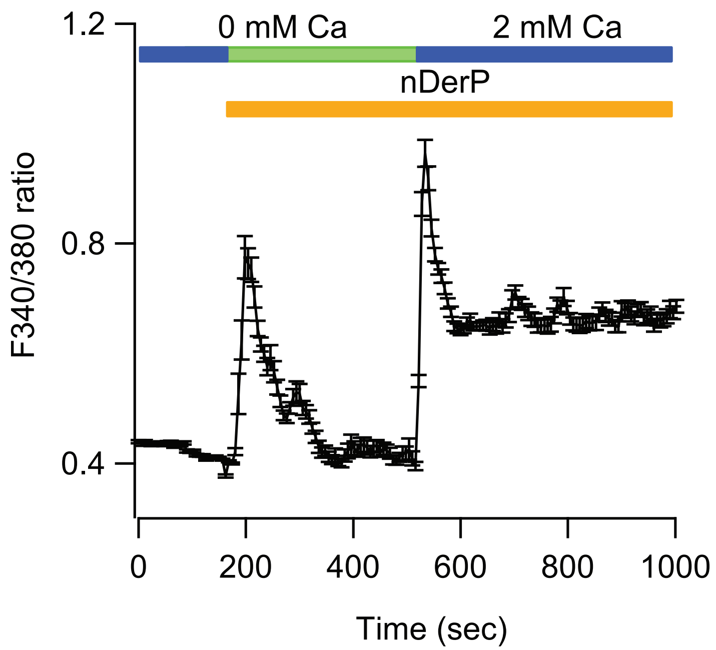

Supplementary Figure S2: House dust mite extract releases intracellular Ca^{2+} stores. Fura-2 imaging of BEAS2b cells showing Ca^{2+} release from intracellular stores following administration of house dust mite extract (nDerP, 18µg/mL) in a Ca^{2+}-free Ringer's solution. Subsequent readdition of extracellular Ca^{2+} (2 mM) evokes store-operated calcium entry.
